# A Standardized Approach to Non-Infectious Disease Cluster Investigations in the U.S. Department of the Air Force

**Published:** 2026-09-20

**Authors:** Amber Britt, Keith Beam, Sarah M. Fryman-Wynkoop, Gregory G. Wolff

**Affiliations:** U.S. Air Force School of Aerospace Medicine (USAFSAM), Epidemiology Consult Service Division (PHR), Defense Centers for Public Health–Dayton, Wright Patterson Air Force Base, OH

## Abstract

The U.S. Air Force School of Aerospace Medicine (USAFSAM) developed a systematic, multi-phased process for receiving, evaluating, and responding to inquiries about potential non-infectious disease clusters, particularly cancer, within the Department of the Air Force. Created in response to a rise in cluster concerns, this process provides a validated, transparent, and objective evaluation framework with 4 distinct phases: 1) establishing lines of communication, 2) feasibility assessment, 3) epidemiological evaluation, and 4) epidemiological investigation. This framework allows a merit-based evaluation for determining if a full epidemiological study is warranted. The framework emphasizes robust risk communication and partnerships to manage expectations and address all concerns with scientific rigor. In 2023 and 2024 this process evinced savings of $6.25 million and 102 months of person-time for the USAFSAM team, ensuring responsible use of public health resources.

## Background and Operational Context

In recent years, the U.S. Department of the Air Force has received an increasing number of inquiries regarding perceived clusters of non-infectious diseases, particularly cancer. The U.S. Centers for Disease Control and Prevention (CDC) defines a disease cluster as “a greater than expected number of the same or etiologically related cases within a specific geography and time period.” ^1^ While identifying a true cluster can inform public health policy, establishing a definitive link to an environmental cause is complicated due to numerous factors. These challenges include long latency periods between exposure and disease onset, population mobility, confounding lifestyle and genetic factors, and the statistical instability inherent in small case counts. ^1^

**What is the impact on readiness and force health protection?**

The process enables a timely, evidence-based response to potential health threats. It ensures public health resources are used responsibly, for scientifically valid investigations, and builds trust within the Air Force community to directly support the health, fitness, and readiness of U.S. Air Force service members.

Historically, investigations of suspected cancer clusters rarely confirm a cause. A 2012 review by Goodman et al. found that only 72 of 576 investigations of suspected cancer clusters were confirmed as having statistical excesses of cases, and just 1 had a definitive, established cause. ^2^ This finding highlights the need for a precise balance between receptiveness to concerns about non-infectious diseases and finite public health resources.

To address these challenges, the U.S. Air Force School of Aerospace Medicine (USAFSAM) established a standardized process, based on CDC guidelines, to objectively investigate potential non-infectious disease clusters. ^1 , 3^ This report describes the USAFSAM process and its demonstrated application and value for the Department of the Air Force, Public Health, and the Department of War.

## Framework

The USAFSAM framework for evaluating suspected disease clusters is a 4-phased, collaborative process, aligned with the CDC’s 4-step framework for cluster investigations. ^1 , 3^ The USAFSAM framework was formalized in 2023 and subsequently codified into a standard operating procedure (SOP). ^4^ The framework involves partner-ships with major commands, leadership, as well as installation public health, bioenvironmental engineering, and aerospace medicine assets. The investigative approach is summarized in the **Figure** .

**FIGURE.**
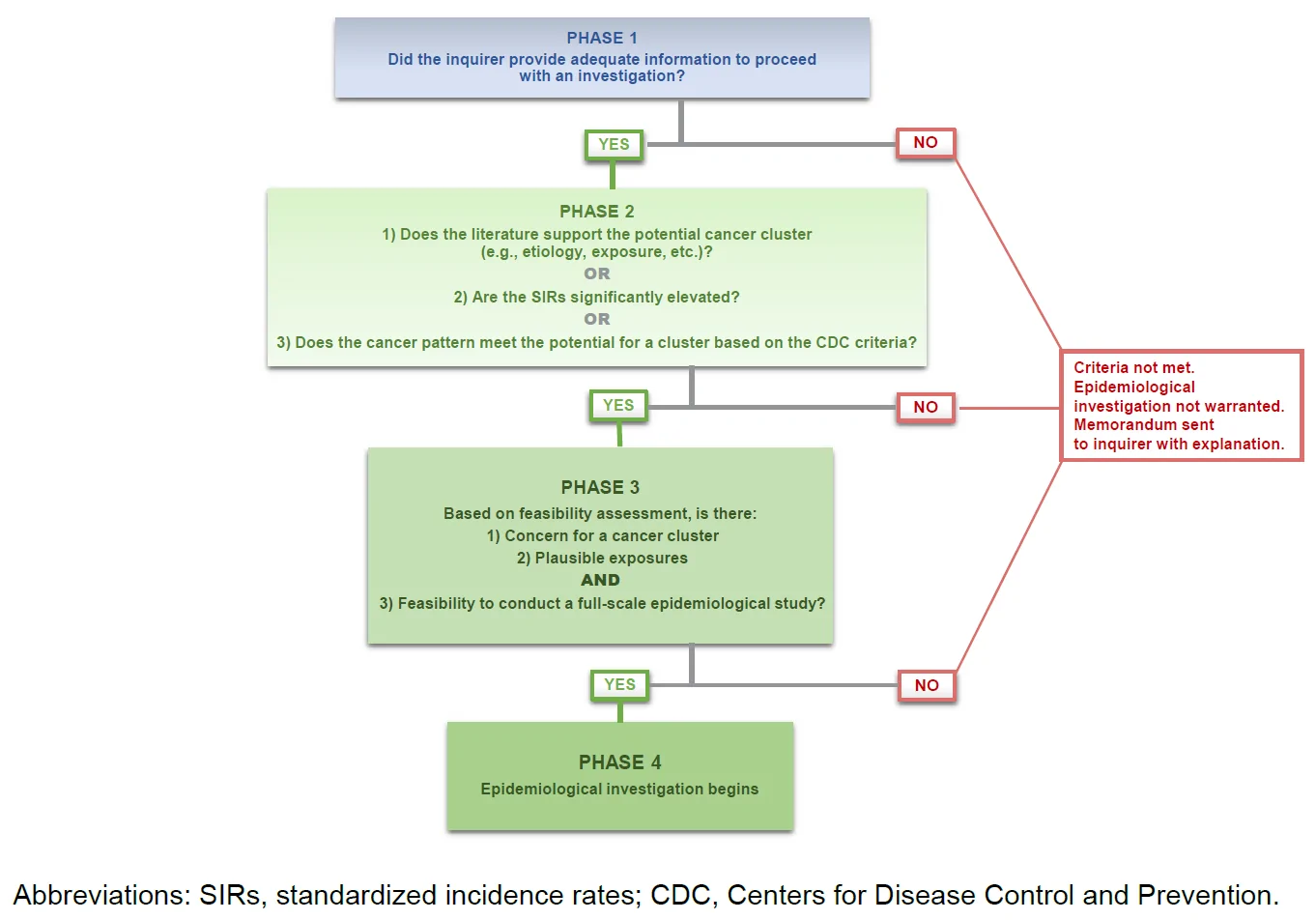
Department of the Air Force Cancer and Non-Infectious Disease Cluster Response Process Phases

### Phase 1: Establishing lines of communication

The initial phase begins when a concern about a potential non-infectious disease cluster is first reported. The primary goal is to gather as much information as possible from the individual or group raising the concern. Information gathered includes details about the types of illnesses, numbers of cases, geographic areas of concern, and periods when illnesses were observed. USAFSAM evaluates the final merit of an inquiry based on the initial validity assessment provided by the installation’s public health and medical assets.

During this phase, it is crucial to establish a trusting relationship with the community by listening empathetically to their concerns and providing clear, consistent communication. Responders also take this opportunity to educate the community about the nature of the disease in question, its known risk factors, and the process for investigating potential clusters. A key decision at the end of this phase is to determine if the reported situation warrants further investigation, based on whether the information provided could plausibly meet the definition of a disease cluster.

To facilitate the assessment, USAF-SAM requests specific information from the inquiring unit, including: a roster or estimated size of the population at risk during the specified timeframe; a line list of affected individuals, including demographics and clinical information (e.g., disease type, date of diagnosis); any observed patterns in the timing of diagnoses or other notable trends, such as cases diagnosed at unusually young ages; and details on specific exposures of concern and known or suspected routes of exposure. A preliminary case definition is constructed to specify the non-infectious disease condition, affected population, location, and period of interest (i.e., “any cases of prostate cancer in active duty maintenance personnel located at building X at base X from 2005-2015”).

To proceed with phase 2, the assessment requires either an adequate number of etiologically linked cases (verified by a power calculation assessment, generally 16 or more cases) or a determination that the number of cases is concerning given the population size. ^5^

### Phase 2: Feasibility assessment

Once it is determined that a concern warrants further attention, the next step is a feasibility assessment to determine if a statistically significant excess of cases exist. This is a preliminary statistical analysis that compares the numbers of reported cases in the community to the numbers of cases expected based on rates in a larger comparison population, such as the state. During this phase, an SOP is employed that specifies which activities can legally be conducted by USAFSAM prior to an epidemiological investigation. As a public health authority operating under HIPAA (Health Insurance Portability and Accountability Act), USAFSAM is legally permitted to review the minimum necessary protected health information (PHI) solely to prepare research protocols, provided no PHI is removed or stored from its original source. These preparatory activities are strictly limited to data validation and feasibility assessments and must be concluded before a formal study begins, under institutional review board (IRB) approval. These preparatory activities are needed to determine the merit of further investigations and prioritize resources. The preparatory activities designation, in conjunction with a public health authority determination, allows USAFSAM to augment self-reported case counts from the inquiring unit by utilizing Air Force medical and personnel databases. These activities, in conjunction with the inquirers case ascertainment, provide appropriate estimates of case capture required for phase 2.

USAFSAM epidemiologists validate case numbers and calculate standardized incidence ratios (SIRs) or standardized mortality ratios (SMRs) by comparing the observed cases to the expected number in a similar reference population (e.g., state specific cancer registries). An SIR or SMR greater than 1.0 indicates that there are more cases or deaths than expected. This phase requires careful definition of the study population, geographic boundaries, and period for the analysis. The findings of this assessment help to determine if a more in-depth investigation is scientifically justified.

To proceed with phase 3 of the assessment, requirements for advancing from phase 1 to phase 2 must be met. Additionally, calculated SIRs or SMRs must provide evidence of elevated incidence or mortality in the study population.

### Phase 3: Epidemiological evaluation

This phase serves as the critical decision point for determining if a full-scale epidemiological investigation is warranted and feasible. Using the data gathered in phase 2’s feasibility assessment, USAF-SAM conducts a merit-based evaluation to synthesize the findings and decide on the path forward. The primary goal is to assess whether a more resource-intensive study is scientifically justified, has a reasonable chance of success, and could contribute to public health knowledge or policy.

The evaluation considers several key factors. An investigation is typically merited if there is a statistically significant excess of a specific disease, a confirmed exposure to a known environmental or occupational hazard, and a large enough number of cases (generally 16 or more) to provide adequate statistical power. ^5^ The potential for the findings to affect Air Force policy or Department of Veterans Affairs (VA) benefits are also key considerations.

Conversely, a full investigation is generally not pursued if the number of cases is too small for a meaningful statistical analysis, if no statistically significant excess of disease is found, or if there is no probable or biologically plausible environmental exposure. Additionally, if the link between the exposure and the outcome of interest is already well established in scientific literature, a new study may not be undertaken. For instance, there is a well-established association between Parkinson’s disease and military toxic exposures, a presumptive service connection recently expanded by the PACT Act (Sergeant First Class Heath Robinson Honoring our Promise to Address Comprehensive Toxics Act) of 2022. This recognition has driven largescale funding through Congressionally Directed Medical Research Programs and the VA, meaning foundational descriptive epidemiological studies by individual services are less important at this point. ^6^ The ultimate decision from this phase is to either proceed to a full epidemiological investigation (phase 4) or to conclude the inquiry and communicate the findings and rationale to concerned parties.

To proceed with phase 4 of the assessment, requirements for moving from all previous phases must be met. Additionally, a confirmed exposure to a known environmental or occupational hazard must exist for the non-infectious disease of interest. Furthermore, the disease cluster investigated must affect Air Force or Public Health policy or contribute to scientific knowledge. Lastly, the investigation must be novel, not duplicating previously established exposure and disease relationships, nor duplicating the work of others.

### Phase 4: Epidemiological investigation

The final phase is the formal epidemiological investigation, which is a comprehensive scientific study designed to test the hypothesis developed in the previous phase. This is the most resource-intensive part of the process and is only undertaken when strong evidence suggests that the study will have merit, may affect policy, or contribute to scientific knowledge. This phase requires an IRB determination to access and utilize cancer registry data to include the Department of Defense Central Cancer Registry (DoDCCR), VA Central Cancer Registry (VACCR), or any state or territorial registries.

Throughout an investigation it is critical to communicate to the community that even a full-scale investigation may not identify a definite cause, due to the complexity of disease causation and the challenges of accurately assessing past exposures. Ultimately, all findings of an investigation are shared with all interested parties, to ensure transparency, address public health concerns, and promote healthy behaviors.

## Operational Outcomes

By applying the multi-phased approach to incoming consultations, USAFSAM was able to triage which inquiries required full-scale epidemiological investigations versus those that could be addressed through other means, such as an initial email response, formal memorandum, or public meeting, because they did not meet the necessary criteria for a full study. From 2023-2024, the framework was applied to 8 representative consultations, to assess their need for full epidemiological investigation **(Table)** . Two consultations warranted full epidemiological studies, but the remainder did not warrant further investigation: 3 were resolved at phase 1, 2 were resolved at phase 2, and 1 was resolved at phase 3. The following 2 consultations are examples that did not warrant a full study.

**TABLE.** Descriptions of Consultations, with Costs and Durations for Completion, January 1, 2023–December 31, 2024

Description of Consultation	Resolution	Disposition	Estimated Cost	Estimated Time (mo.)	Cost Avoided	Time Saved (mo.)
Totals					$6,250,000	102
Non-cancer concern, base A	Resolved, phase 3	Conference call	$750,000	9	$562,500	9
Non-cancer concern, base B	Resolved, phase 1	Memorandum	$750,000	9	$562,500	9
Cancer concern, base C	Resolved, phase 1	Email response	$1,500,000	24	$1,500,000	24
Cancer concern, population D	Resolved, phase 2	Memorandum, talking points	$1,500,000	24	$1,500,000	24
Cancer concern, base E	Resolved, phase 2	Memorandum, public meeting	$1,500,000	18	$1,500,000	18
Cancer concern, base F ^a^	Phase 4 study	Memorandum, Congressional briefing	$750,000	6	N/A	N/A
Cancer concern, population G ^a^	Phase 4 study	Memorandum, public meetings	$1,500,000	24	N/A	N/A
Cancer concern, base H	Resolved, phase 1	Email response	$1,125,000	18	$1,125,000	18

a Consultation completed all 4 phases, resulting in a comprehensive epidemiological investigation.

In July 2023, an inquiry was received from an Air Force Reserve unit about a perceived elevated cancer risk among its personnel, located on a closed Air Force base designated as a Superfund site. The unit provided a list of 18 individuals diagnosed with different types of cancer over a 10-year period. USAFSAM proceeded to investigate the potential cluster using phases 1 and 2 of the new procedure. By calculating SIRs and comparing the unit’s data to state-specific cancer rates, USAFSAM epidemiologists determined that the observed 18 cases were well below the expected number of approximately 50 cases for that population. Since the analysis did not show a greater than expected number of cancers, and the reported cancers were not etiologically related, a full epidemiological investigation was not warranted, and the consultation was resolved at phase 2. The results and rationale were formally communicated to unit leadership and subsequently shared with all personnel in a public meeting, evincing the process’s effectiveness in providing a timely, data-driven, and transparent response.

In September 2023, USAFSAM received an inquiry regarding concerns of a potential amyotrophic lateral sclerosis (ALS) cluster associated with a specific residential area at an Air Force installation from 2004 through 2007. The initial investigation included collaboration with national environmental and disease experts, a review of historical environmental surveillance data, and an examination of individual medical records. Epidemiologists determined that the total number of ALS cases was too low to conduct a reliable statistical analysis or meet the threshold required to advance to phase 2. Additionally, because there are no known environmental triggers for ALS, and none of the individuals were diagnosed while stationed at the base, no scientifically supported correlations could be drawn. As a result, a full epidemiological investigation was not warranted, and the consultation was resolved at the end of phase 1. Findings were formally communicated to the unit, along with educational materials and guidance encouraging affected military families to participate in the national ALS registry.

The total cost avoidance of the 6 studies not warranting a full-scale investigation was $6,250,000 and yielded a time savings of 102 months. These figures were derived from the estimated duration of complex studies multiplied by the salaries of the required investigative team (e.g., physicians, epidemiologists, data managers). The analysis of these savings is detailed in the **Table** .

The standardized process was successfully disseminated after being presented to public health professionals at an Air Force conference in February 2025, and the SOP was published on an Air Force website in April 2025. In addition, the process was presented at the Military Health System Research Symposium in August 2025, generating international interest with a health protection delegation from a partner nation. The framework’s practical value was again demonstrated in August 2025 when a Public Health officer independently applied the procedure to evaluate a potential testicular cancer cluster, an assessment later validated by USAFSAM. This successful field application underscores the procedure’s effectiveness in providing timely, evidence-based, and objective evaluations at the installation level.

## Public Health Implications

The successful implementation and evaluation of this standardized process provide the Air Force with a transparent, validated, and resource efficient framework. The results demonstrate that by systematically triaging inquiries, the Air Force can achieve significant cost and time savings, optimizing finite public health resources. This multi-team approach ensures that concerns are evaluated objectively and that full-scale investigations are directed toward inquiries with the greatest scientific merit and potential public health importance.

The challenges inherent in these investigations, as outlined by the CDC, cannot be overstated. ^1 , 3^ Proving causation is exceptionally difficult due to long latency periods, population mobility that complicates exposure assessments, absence of completed exposure pathway, and numerous confounding variables such as diet, heredity factors, and outside environmental exposures. ^1 , 3^ In Goodman et al.’s study, out of 576 cancer cluster investigations, only 1 had a clearly identified cause, underscoring the rarity of establishing a definitive link. ^2^

The Air Force’s process acknowledges these scientific hurdles by managing expectations early and focusing on statistical evaluation before committing to a costly and time intensive full-scale etiological investigation. The framework’s emphasis on partnership and risk communication is critical. Cancer is a common and emotionally charged disease. By communicating the scientific process and results transparently, leadership can address concerns directly and build trust, even when a full investigation is not warranted. This validated framework ensures all concerns are evaluated equitably and scientifically, prioritizing the health and safety of the Air Force community while maintaining responsible resource stewardship.

## References

[1] Abrams B , Anderson H , Blackmore C , et al . Investigating suspected cancer clusters and responding to community concerns: guidelines from CDC and the Council of State and Territorial Epidemiologists . MMWR Recomm Rep . 2013 ; 62 ( rr-08 ): 1 - 24 . Accessed Jul. 28, 2026 . https://www.cdc.gov/mmwr/preview/mmwrhtml/rr6208a1.htm 24067663

[2] Goodman M , Naiman JS , Goodman D , LaKind JS . Cancer clusters in the USA: what do the last twenty years of state and federal investigations tell us? Crit Rev Toxicol . 2012 ; 42 ( 6 ): 474 - 490 . doi: 10.3109/10408444.2012.675315 PMC3408895 22519802

[3] Centers for Disease Control and Prevention . Guidelines for examining unusual patterns of cancer and environmental concerns . Unusual Patterns of Cancer, the Environment, and Community Concerns . U.S. Dept. of Health and Human Services . Updated May 6, 2026 . Accessed Jul. 28, 2026 . https://www.cdc.gov/cancer-environment/php/guidelines/index.html

[4] U.S. Air Force School of Aerospace Medicine, Defense Centers for Public Health–Dayton . Public Health Authority and Standard Operating Procedure for Activities Preparatory to Research . Dept. of the Air Force, U.S. Dept. of War . Apr . 2024 .

[5] Cancer Surveillance Branch, Division of Cancer Prevention and Control, National Center for Chronic Disease Prevention & Health Promotion; Surveillance Research Program, Division of Cancer Control and Population Sciences, National Cancer Institute . United States Cancer Statistics Restricted Access Data Set: Data Dictionary and Data Standards, 2015 November Data Submission . Centers for Disease Control and Prevention, U.S. Dept. of Health and Human Services . Jun . 2016 . Accessed Mar. 13, 2026 . https://www.cdc.gov/rdc/data/b1/uscs_rdc_datadictionary_2016.pdf

[6] Congressionally Directed Medical Research Programs . Parkinson’s Research Program: Advancing Parkinson’s Disease Research Toward Treatments . U.S. Dept. of War . Jul . 2023 . Accessed Jul. 28, 2026 . https://cdmrp.health.mil/prp/pbks/prppbk2023.pdf

